# Drug discovery with an RBM20 dependent titin splice reporter identifies cardenolides as lead structures to improve cardiac filling

**DOI:** 10.1371/journal.pone.0198492

**Published:** 2018-06-11

**Authors:** Martin Liss, Michael H. Radke, Jamina Eckhard, Martin Neuenschwander, Vita Dauksaite, Jens-Peter von Kries, Michael Gotthardt

**Affiliations:** 1 Neuromuscular and Cardiovascular Cell Biology, Max Delbrueck Center for Molecular Medicine in the Helmholtz Association, Berlin, Germany; 2 DZHK (German Centre for Cardiovascular Research), partner site Berlin, Berlin, Germany; 3 Screening Unit, Leibniz-Institut für Molekulare Pharmakologie, Berlin, Germany; Iowa State University, UNITED STATES

## Abstract

Diastolic dysfunction is increasingly prevalent in our ageing society and an important contributor to heart failure. The giant protein titin could serve as a therapeutic target, as its elastic properties are a main determinant of cardiac filling in diastole. This study aimed to develop a high throughput pharmacological screen to identify small molecules that affect titin isoform expression through differential inclusion of exons encoding the elastic PEVK domains. We used a dual luciferase splice reporter assay that builds on the titin splice factor RBM20 to screen ~34,000 small molecules and identified several compounds that inhibit the exclusion of PEVK exons. These compounds belong to the class of cardenolides and affect RBM20 dependent titin exon exclusion but did not affect RBFOX1 mediated splicing of FMNL3. We provide evidence that cardenolides do not bind to the RNA interacting domain of RBM20, but reduce RBM20 protein levels and alter transcription of select splicing factors that interact with RBM20.

Cardenolides affect titin isoform expression. Understanding their mode of action and harnessing the splice effects through chemical modifications that suppress the effects on ion homeostasis and more selectively affect cardiac splicing has the potential to improve cardiac filling and thus help patients with diastolic heart failure, for which currently no targeted therapy exists.

## Introduction

In developed countries heart failure (HF) keeps the top spot in mortality statistics and although prevention and therapy have continuously been improved over the past 30 years, the prevalence of heart failure remains high [1]. Multiple environmental and genetic factors contribute to the development of heart failure. This includes age, sex, diabetes, kidney disease, inflammation, and mutations in sarcomeric proteins such as titin and splice factors such as RBM20, a regulator of titin based stiffness [2]. The classification into systolic versus diastolic heart failure relates to the underlying pathophysiology with reduced ejection fraction in systole (HFrEF) or inefficient filling of the ventricle in diastole with preserved ejection fraction (HFpEF), respectively [3]. Half of all patients with HF belong to the latter group, which is heterogeneous and poorly characterized [4].

Systolic heart failure has been researched intensively, resulting in efficient therapies. This is unlike diastolic heart failure, where patients do not have access to targeted treatment options [5]. As genetic defects in RBM20 increase the size of titin isoforms, reduce cardiomyocyte resting tension, and improve diastolic function [2,6,7], while shortening titin’s elastic N2B, PEVK, or IG regions impair diastolic filling [8–10], RBM20 could serve as a therapeutic target in diastolic dysfunction [11–13]. Accordingly, we evaluated if titin isoform expression could be adapted as a readout for a cell based screen of RBM20 inhibitors. Here, we describe the basis of our splice reporter screen and the identification of cardenolides as efficient inhibitors of RBM20-mediated alternative splicing of titin. We evaluate their mode of action, which relates to an effect on expression of RBM20 cofactors in a cell based system.

Although modifications that improve bioavailability and reduce side effects are needed before derivatives can be applied *in vivo*, the efficiently increased exon inclusion in cell culture, suggests cardenolides as a new class of compounds that could be adapted to improve the therapy of diastolic dysfunction.

## Materials and methods

### Experimental design

The objective of our study was to identify and evaluate small molecules with the potential to modulate alternative splicing via an unbiased chemical high-throughput screening approach. Using transient transfection of a dual luciferase splicing reporter assay (DLR-assay) in HEK293.EBNA cells and a RBM20 expression construct, we investigated >34.000 substances for their effect on alternative exon exclusion from our RBM20 dependent titin PEVK4-13 splicing reporter. For the primary screen (pilot screen), each compound was tested at 10 μM (single point) and compared to an untreated control. Positive hits leading to differential exon usage (exceeding three standard deviations) were picked for independent validation in 9 serial 2-fold dilutions starting at 20 μM. Compounds that produced a concentration dependent effect were validated manually in the 96-well format and cytotoxicity was evaluated. To minimize the detection of false positives, we excluded compounds with structural homology to each luciferase’s natural substrate. To determine the EC_50_ of candidates we calculated dose response curves using a non-linear least-squares fitting routine available within the GNUPLOT package (accessible online: http://ic50.tk/index.html). We evaluated the effect on splice activity on the RNA level by RT-qPCR of isoforms generated from a titin minigene containing TTN Ex241-43 (corresponding to TTN I-band exon I96-98) in the presence of the potential splice modulators. Compounds that produced a concentration depend effect in the DLR-assay and altered the alternative transcript levels were regarded as validated alternative splicing modulators.

### Reagents

The compound library used for high-throughput screening contained 34144 compounds. 21824 derived scaffolds from the World Drug Index (WDI) of which 16544 were chosen based on diversity, 5280 with an emphasis on solubility and 4576 carboxylate/ketone and amine fragments.

4224 compounds were obtained from the ARTCHEM-library (ART-CHEM GmbH, Berlin, Germany) and 4312 compounds were donated to the FMP-Berlin by academic researchers. Compounds were dissolved in DMSO and diluted 1:1 in acetonitril/water to a final concentration of 25 μM and filtered before use. Purity was determined according to the UV absorbance at 254 nm using LC-MS. Compounds for manual validation were obtained from Sigma-Aldrich, EMD Millipore or purchased via MolPort at the highest available purity and diluted in DMSO (Sigma-Aldrich Chemie GmbH, Munich, Germany).

### Cell culture experiments

Human embryonic kidney (HEK293.EBNA) cells (Life Technologies GmbH, Darmstadt, Germany) were maintained in high glucose Dulbecco’s modified eagle medium (DMEM) supplemented with 10% fetal bovine serum (Sigma-Aldrich Chemie GmbH, Munich, Germany [F7524, Lot#124M3337]) and 1% penicillin/streptomycin 10,000 U/mL (Gibco by Life Technologies GmbH, Darmstadt, Germany).

For transfection 25000 cells/well were seeded on 96-well Nunc F96 MicroWell™ plates (Life Technologies GmbH, Darmstadt, Germany) and transfected with a total of 200 ng of plasmid DNA of which 1 ng was splice reporter plus a corresponding amount of RBM20, RBFOX1 or control plasmid (pcDNA3.1) in a 20x molar excess. To deliver plasmid DNA we used the 40 kDa linear polyethylenimine (Polysciences Europe GmbH, Hirschberg, Germany) at a 1:3 ratio (DNA:PEI40). Cells transfected were at a confluency of 50-60%. Compounds were applied 24 h post-transfection at a final DMSO concentration of 1%. Luciferase activity was measured 60 hours post-transfection using the Dual-Luciferase® Reporter Assay System (Promega GmbH, Mannheim, Germany) on an Infinite® M200 Pro (TECAN, Maennedorf, Switzerland) plate reader. Ratios of firefly to renilla luciferase activity were normalized to the control (pcDNA3.1) without the splicing factor expression construct. Cell viability was measured 60 h post-transfection using a resazurin based staining of metabolically active cells (PrestoBlue®, Life Technologies GmbH, Darmstadt, Germany).

### Compound library screening

5000 HEK293.EBNA cells were seeded in clear bottom, white 384-well plates (Corning by Sigma-Aldrich Chemie GmbH, Munich, Germany) using an automated cell dispenser (Biotek, Bad Friedrichshall, Germany). 24 h after cell seeding the expression plasmids were delivered using a Freedom EVO® robotic workstation (TECAN, Maennedorf, Switzerland) equipped with a MultiChannel Arm™. Cells were transiently transfected with 45 ng plasmid DNA (2 ng DLR and 40 ng RBM20 or 24 ng of the negative control pcDNA3.1 ad 45 ng with bacterial expression plasmid pBSSK). Compounds were added to the plates the following day using the same robotic platform. Luciferase activity was determined using Dual-Glo^®^ Luciferase Assay System (Promega GmbH, Mannheim, Germany) on an Infinite® M1000 Pro (TECAN, Maennedorf, Switzerland) plate reader around 60 hours post-transfection. Assay robustness was evaluated by calculation z’ scores using an online tool provided by the Screening Unit at FMP Berlin (http://www.screeningunit-fmp.net/tools/z-prime.php).

### Surface plasmon resonance

Surface plasmon resonance spectroscopy was used to study the binding of digitoxin and digitoxigenin to RBM20. The RRM domain of RBM20 was cloned into pGEX-6P-1 bacterial expression plasmid and expressed in E. coli Rosetta™ 2 (DE3) (Novagen by Merck KgaA, Darmstadt, Germany). After purification of the recombinant protein using glutathione-sepharose (Sigma-Aldrich Chemie GmbH, Munich, Germany) the purified fraction was dialyzed o/n in reaction buffer (20 mM HEPES pH7, 200 mM NaCl, 1 mM MgCl2, 10% glycerol). The surface plasmon resonance study (SPR) was performed on a Biacore T100 (GE Healthcare Europe GmbH, Freiburg, Germany) using amine coupling. Chip surface was activated with 0.2 M EDC/0.4 M NHS. 30 μg of human serum albumin (HSA) (positive control) or RBM20-RRM were linked to the activated chip surface at pH 4.5 in 10 mM sodium acetate.

### Protein analysis

For immunodetection, HEK293.EBNA samples were harvested by rinsing them of the cell culture substrate using PBS at 37°C and subsequently used for downstream processing or snap frozen in liquid nitrogen. For protein extraction samples were suspended in RIPA buffer (50 mM Tris pH 8.0, 150 mM NaCl, 1% NP-40, 0.25% sodium deoxycholate, 1.0 mM EDTA; 0.1% SDS) and lysed via sonication with 20 bursts at 70% energy using a VialTweeter (Hielscher Ultrasonics GmbH, Teltow, Germany). Protein concentration was estimated after 20’ precipitation of cell debris at 13,000xg for 20 min at 4°C using BCA assay (Life Technologies GmbH, Darmstadt, Germany [23225]). Samples were kept cool at any time. Lysates were separated by SDS-PAGE [14] and blotted on PVDF membranes [15]. Blotted antigens were probed with mouse anti-c-myc 9E10 ([M5546], Sigma-Aldrich Chemie GmbH, Munich, Germany) tag antibody at 1:2000 and detected using a sheep anti-mouse IgG-HRP (GE Healthcare Europe GmbH, Freiburg, Germany [NA9310V]) conjugate 1:5000 follow by visualization with AceGlow™ (VWR PEQL37) on a FUSION FX7 CCD camera system (Vilber Lourmat Deutschland GmbH, Eberhardzell, Germany).

### Real-time quantitative PCR

Total RNA from HEK293.EBNA cells was extracted using RNeasy Micro Kit (Qiagen, Hilden, Germany) according to the manufacturer’s instructions. 2 μg of total RNA were reverse transcribed using the High-Capacity RNA-to-cDNA Kit (Life Technologies GmbH, Darmstadt, Germany). Quantitative RT-PCR was performed using SYBRGreen master mix (Applied Biosystems by Thermo Fisher Scientific Inc., USA) on a 7900 HT RT-cycler (Applied Biosystems by Thermo Fisher Scientific, Inc., USA). Primer sets are listed in S2 Table. To quantification gene expression, we used the ΔΔC_T_ method normalized to 18S or to the alternative transcripts generated from the splicing reporter. PSI values were calculated as ratio of unspliced and spliced reporter transcript. For conventional PCR we used primer sets listed in S1 Table.

### Expression profiling

Expression profiling data (Gene Expression Omnibus—GSE36058) was analyzed using AltAnalyze [16]—gene expression cut-off = 1.0. Gene expression differences of digitoxin treated HEK293 cells were expressed as fold-changes compared to the DMSO treated controls.

### Statistical analysis

Data are displayed as means ±SD. Groups were compared with unpaired, two-tailed t-tests, one-way-, or two-way ANOVA (with Tukey post-test) as appropriate using Prism 5.0 (GraphPad Software, Inc., La Jolla, USA). The significance is indicated as P < 0.05 (*), P < 0.01 (**), P < 0.001 (***). The number of biological replicates (N) is indicated in each figure legend.

## Results

### A dual luciferase splicing reporter assay for high-throughput compound library screen

**Fig 1:**
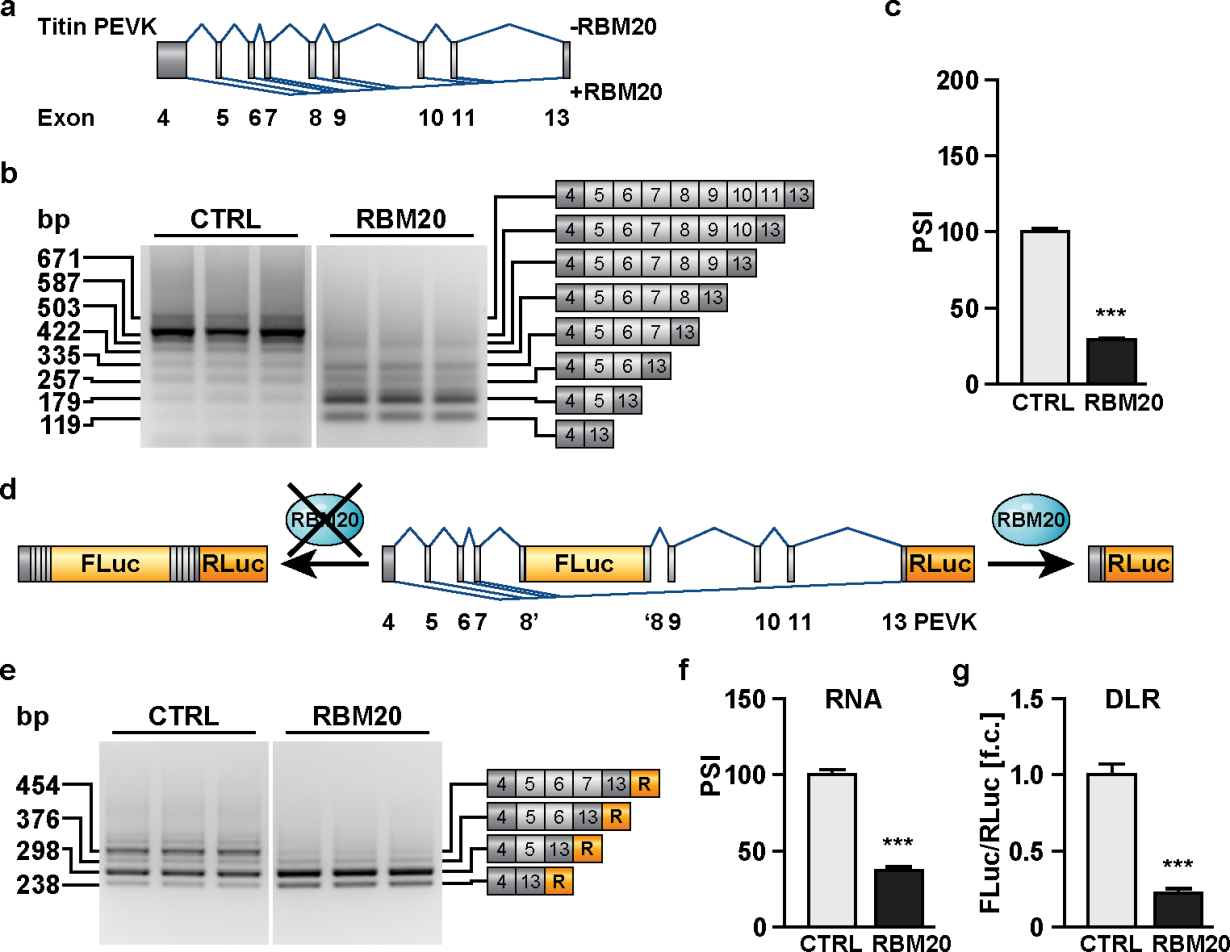
Dual luciferase splicing reporter assay (DLR assay) to identify splicing modulators. **(a)** Minigene containing titin PEVK exons 4–13. Boxes indicate exons, blue lines mature transcripts generated with and without RBM20. **(b)** PCR products of alternative transcripts produced from the PEVK minigene by RBM20. RBM20 increases the amount of transcripts lacking all alternative exons or only retaining alternative exon 5. **(c)** Quantitative PCR to relate inclusion of PEVK exon 8 to the constitutive exon 13 presented as percent spliced in (PSI). RBM20 reduces inclusion of exon 8 by ~70% (N = 3). **(d)** Dual luciferase splicing reporter with firefly luciferase (FLuc) integrated into exon 8 and renilla luciferase (RLuc) downstream of exon 13. The FLuc/RLuc ratio reflects the inclusion ratio of alternative exon 8. **(e)** RBM20 shifts alternative splicing of the dual luciferase reporter (DLR) construct to exclude all alternative exons. **(f, g)** Quantitative PCR (N = 3) and FLuc/RLuc activity (N = 8) produce similar readouts with increased sensitivity of the luciferase based assay. ***P<0.001 versus CTRL (Dunnett’s post-test). Data are presented as mean ±SD.

To evaluate splice active compounds, we generated two dual luciferase reporter constructs using the cytomegalovirus (CMV) promoter to drive transcription from the titin PEVK or immunoglobulin region (PEVK4-13 and I96-98). We have previously shown that both regions are regulated by RBM20 [2]. The native PEVK4-13 minigene contains the three constitutive exons (PEVK4-5 at the 5’-end and PEVK13 at the 3’-end) as well as six alternative exons (PEVK6-11) (Fig 1A) that are repressed by RBM20 in cells (S1A and S1B Fig). The native I96-98 minigene contains the alternative I-band exon I97 and is flanked by the constitutive exons I96 at the 5’ and exon I98 at the 3’-end (S1C Fig). Transient co-transfection of HEK293 cells with the minigenes and the CMV-driven RBM20 expression plasmid resulted in the exclusion of alternative exons in both reporters as determined by RT-PCR and RT-qPCR. RBM20 expression favored the small isoforms containing only PEVK exon 4, 5 and 13 or exon 4 and 13 (Fig 1B) or I-band exons I96 and I98, respectively (S1D Fig). Exon exclusion was equally efficient for both minigenes at ~70% (Fig 1C and S1E Fig).

To facilitate high-throughput screen with a sensitive reporter that is not affected by background fluorescence of the compounds or cell system, we developed dual luciferase splicing reporter (DLR) based on the established minigenes: We integrated firefly luciferase (FLuc) into PEVK exon 8 or I97 and renilla luciferase (RLuc) at the 3’-end of the PEVK exon 13 or I-band exon I98 maintaining the open reading frame (Fig 1D and S1F Fig). Transcripts generated from the PEVK DLR in the presence of RBM20 contain exon 4, 5 and 13 as well as exon 4 and 13 only (Fig 1E). In the I96-98 DLR only I96 and I98 remain (S1G Fig). The reduced ratio of FLuc vs RLuc corresponds to an increased splicing activity in both reporters. On the mRNA level, RBM20 dependent exon exclusion in the I96-98 DLR is less efficient compared to the native minigene with 80% vs. 50% exon inclusion (S1H and S1I Fig). In contrast, the native PEVK reporter is more responsive than the Ig Reporter (30% vs. 50% exon inclusion) and the inclusion of the firefly luciferase increases efficiency to 20% (Fig 1F and 1G). As the readout of luciferase versus mRNA levels exaggerates differences by >10% for RBM20 and the PEVK reporter, the DLR-assay not only faithfully captures RBM20 dependent splice activity, but provides a more robust readout than mRNA level analysis of RBM20 dependent TTN splicing. Accordingly, we used the PEVK DLR assay for our screen.

**Fig 2:**
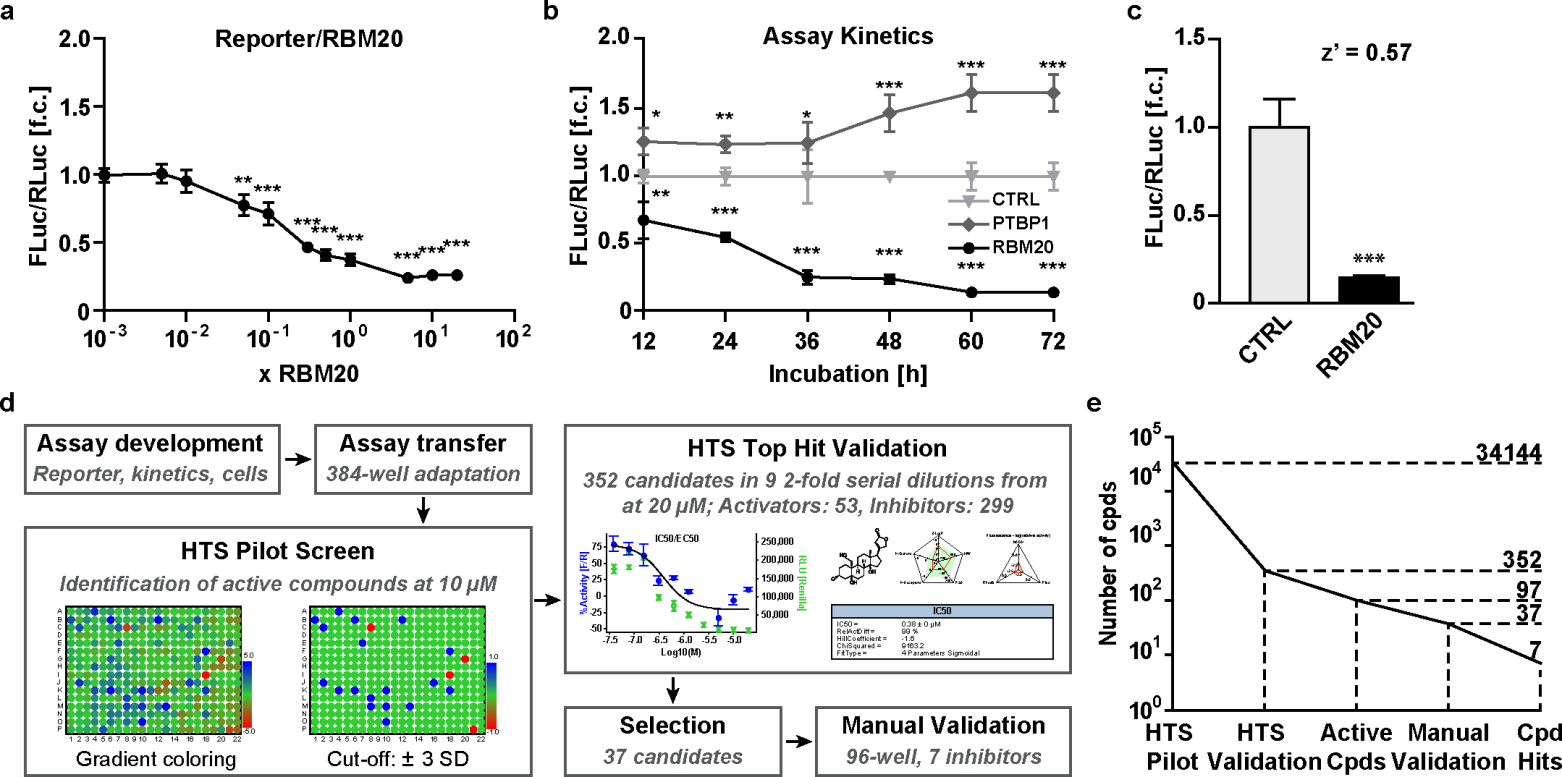
Identification and validation of splice active compounds by a semi-automated high-throughput screen. **(a)** Optimization of the splicing reporter to RBM20 ratio by co-transfection of HEK293 cells. The assay is saturated at a 5-fold excess of RBM20 (N = 8). **(b)** Assay kinetics with maximum effect after 60 hours of incubation (N = 8). Polypyrimidine Tract Binding Protein 1 (PTBP1) served as negative control not leading to exon exclusion of the splice reporter. **(c)** Assay suitability for a high-throughput approach—z’ values >0.5 are adequate. **(d)** Screening strategy to identify splice active compounds. The DLR assay was adapted to the 384-well format followed by the pilot screen with >34,000 compounds at 10 μM. Potentially active compounds were re-evaluated in 9 serial dilutions. Resulting candidates were validated manually in the 96-well format leading to the identification of 7 inhibitors that belong to the group of cardenolides. **(e)** Number of compounds (cpds) passing the different steps of the screening procedure. *P<0.05, **P<0.01, ***P<0.001 versus CTRL (Dunnett’s post-test). Data are presented as mean ±SD.

To adapt the DLR assay to the 384-well format and optimize assay conditions, we adjusted the total amount of DNA transfected, ratio of reporter to RBM20 expression plasmid, number of cells per well, and incubation time (Fig 2A and 2B; S2A and S2B Fig). In the semi-automated robotic setup, we obtained a z’score of 0.57 of the positive (RBM20) versus the negative control (pcDNA3.1), which reflects the robustness of the assay (Fig 2C). DMSO concentrations up to 1% had no effect on the readout (S2C Fig), suggesting that the assay setup was well suited for high-throughput compound screen. An illustration of the screening procedure is provided in Fig 2D. The pilot screen served to establish the assay protocol data analysis workflow. Screening a total of 34,144 compounds identified 59 potential activators and 338 potential inhibitors, with FLuc/RLuc ratios >3 standard deviations away from the mean of which 37 inhibitors were manually evaluated and 10 activators. An example plate with a total of 16 hits is provided in S2D Fig. Among the 397 candidates we picked 299 inhibitors and 53 activators and validated them at 9 serial 2-fold dilutions starting at 20 μM. For 97 compounds we found a dose dependent effect on the FLuc/RLuc-ratio. Candidates associated with stable RLuc values versus decreased RLuc activity were prioritized for follow-up, as RLuc values indicate efficient transcription in a healthy cell so that cytotoxicity of the applied substance is less likely. Compounds that interfered with RLuc values only were not considered as candidates. To reduce the number of potential false positives, we excluded compounds with structural homology to natural luciferase substrates. 10 commercially available potential activators were reevaluated manually, but none of them increased RBM20 sctivity. 37 commercially availableinhibitors were reevaluated in the 96-well format with parallel assessment of cytotoxicity of which 7 were reproducibly found to inhibit RBM20-mediated titin splicing (Fig 2E).

### Cardenolides affect RBM20-mediated splicing of the titin-I-band region

**Fig 3:**
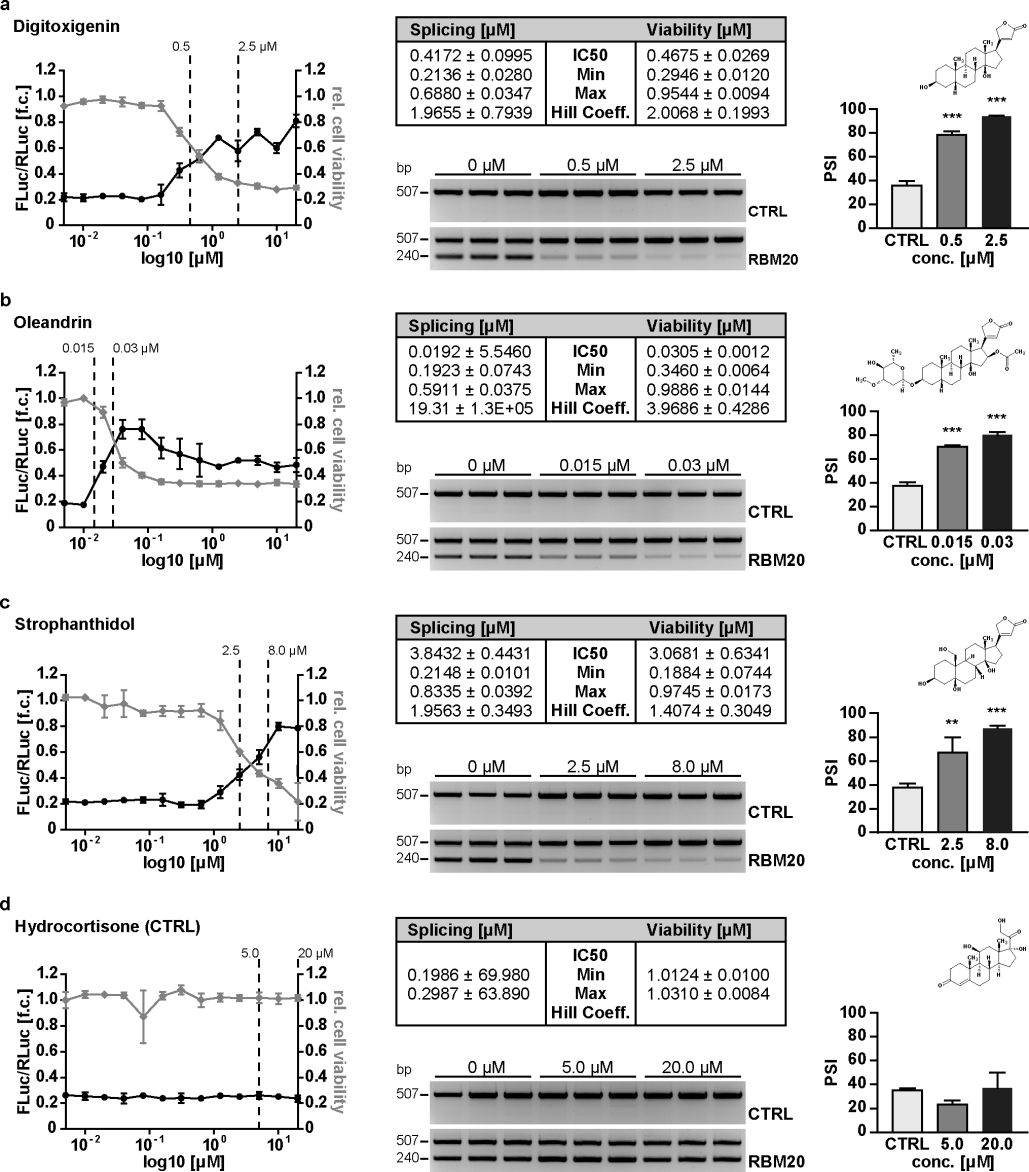
Inhibitors of titin splicing identified by HTS. For each compound concentration dependent activity in the dual luciferase reporter assay and cell viability are plotted. Dashed lines indicate the concentrations used for validation on RNA level. The tables provide kinetic information. Validation by RT-PCR (agarose gel) is quantified by calculating the percent spliced in values (PSI). **(a-c)** Inhibition of alternative splicing was validated manually in the 96-well format using the DLR assay and conventional as well as quantitative RT-qPCR using an independent genomic minigene derived from TTN exons 241-43 (N = 4). Cardenolides efficiently reduce titin splicing by RBM20 with different potency (splicing IC50). The effect on splicing translates to reduced viability of HEK293 cells (IC50 values splicing vs. viability). **(d)** The steroid hydrocortisone does not interfere with splicing activity (N = 4). Compared to the cardenolides it lacks the lactone ring at C17 (chemical structures provided on the right). *P<0.05, **P<0.01, ***P<0.001 versus CTRL (Dunnett’s post-test). Data are presented as mean ±SD.

**Fig 4:**
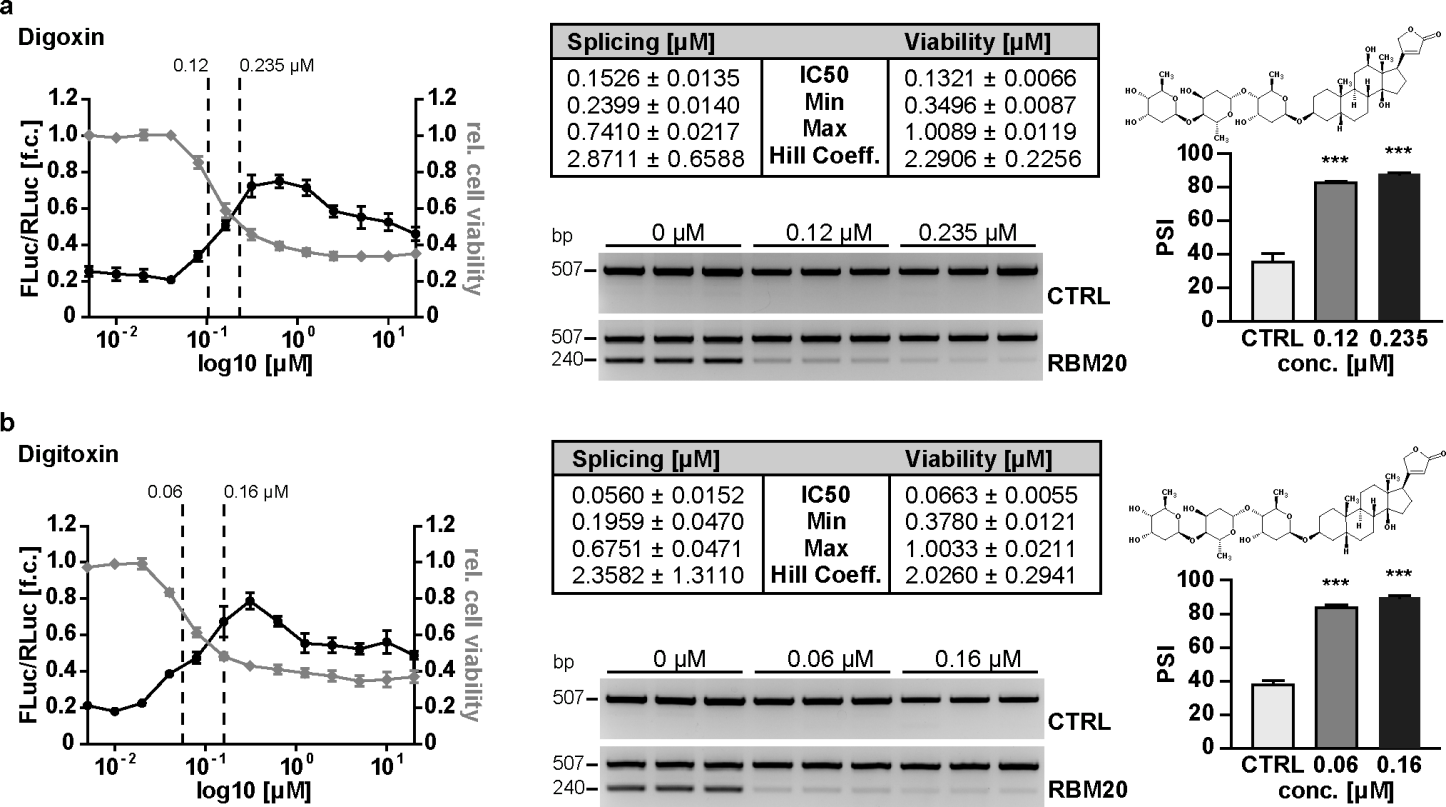
Cardenolides are potent inhibitors of RBM20 dependent titin splicing. Figure layout and panels as described in Fig 3. **(a, b)** Additional cardenolides not present in the original library were selected to evaluate their potency to inhibit RBM20 dependent titin splicing. The structural analogs digoxin and digitoxin both efficiently inhibit RBM20 mediated splicing. (DLR N = 4; RNA N = 3). ***P<0.001 versus CTRL (Dunnett’s post-test). Data are presented as mean ±SD.

Among the compounds identified, the cardenolides stood out for their structural homology and overrepresentation in the group of splicing inhibitors (S1 Table). For 7 cardenolides that were commercially available, we used 13 serial dilutions to evaluate the inhibitory effect (reduced splicing: FLuc/RLuc ratio) and toxicity (reduced transcription: RLuc activity). All compounds were splice active with the half maximal inhibitory concentration (IC50 splicing) in the nano- to micromolar range (Fig 3 and S3 Fig). The cardenolides affect alternative splicing rather than enzymatic activity: RNA isoform expression as determined by both RT-PCR and luciferase reporter activity were affected at the same range of concentrations (Fig 3). Oleandrin was the most potent compound with the splicing IC50 at 20 nM (Fig 3B). Hydrocortisone, which shares the steroidal structure with cardenolides but does not contain a carbohydrate or sugar moiety attached to C-3 of the first carbon ring, did not interfere with splicing (Fig 3D). For all cardenolides tested, the therapeutic index (IC50 viability / IC50 splicing) was low (<2). To evaluate if the splice inhibitory effect extends to cardenolides not present in the original library, we tested the most commonly used cardiac glycosides Digoxin and Digitoxin (Fig 4A and 4B), as well as Ouabain (S4 Fig), which had similar effects on splicing and a similar therapeutic index as the cardenolides identified in our screen.

### Specificity and molecular basis of the cardenolide mediated splice effect

**Fig 5:**
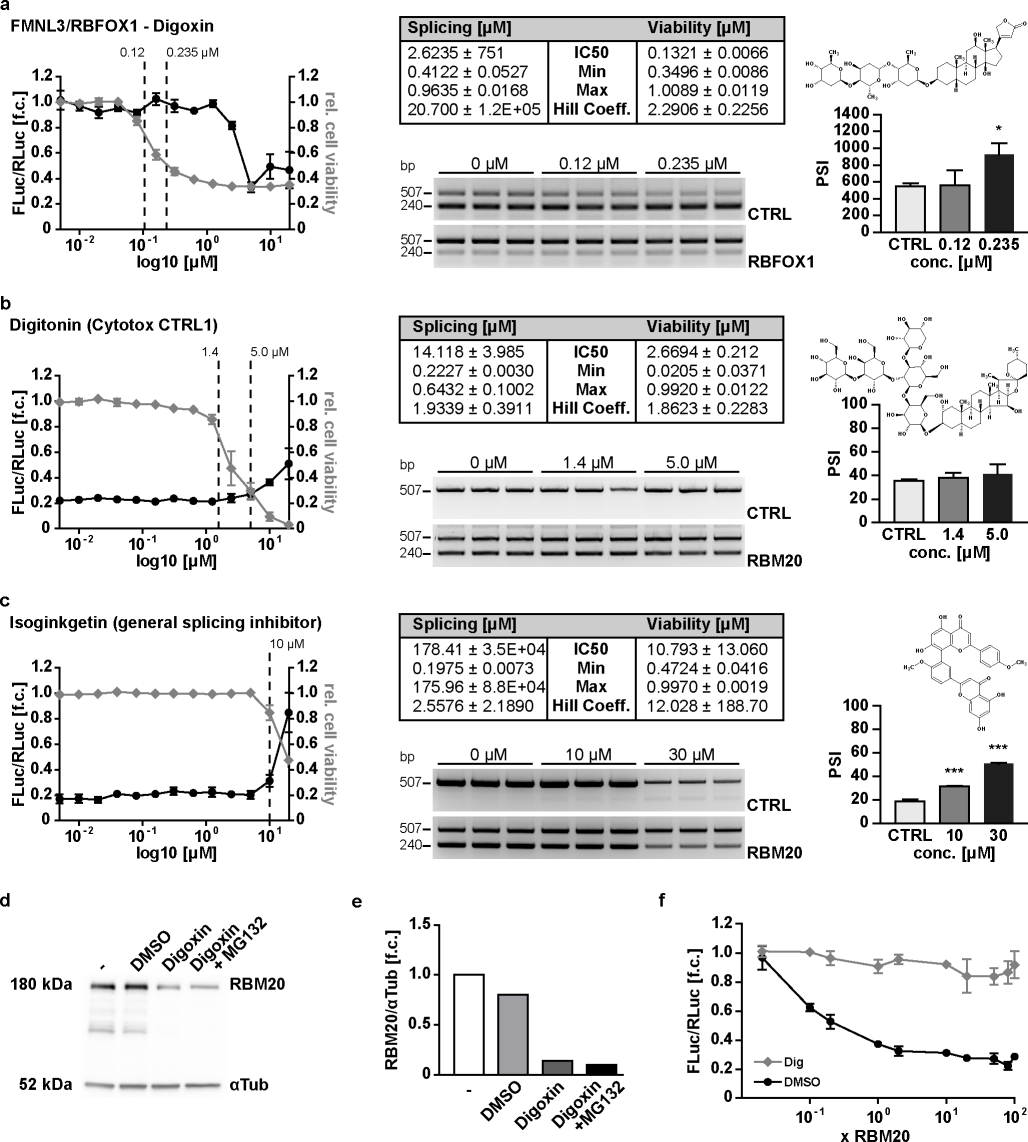
Specificity of the inhibitory effect of cardenolides on RBM20-mediated titin splicing. Figure layout and panels as described in Figs 3 and 4. **(a)** An independent minigene containing FMNL3 exons 25-26 was co-transfected with RBFOX1 and does not respond to digoxin until cell viability is below 30% (N = 3). **(b)** Digitonin does not affect the splice reporter readout until cell viability is below 20%. There is no effect on the mRNA level (N = 3). **(c)** Isoginkgetin treatment reduces alternative splicing and mRNA maturation (reduced PCR product at 507 bp in the control at 30 μM). **(d, e)** Digoxin treatment of co-transfected HEK293 cells leads to decreased expression of RBM20, which is not affected by addition of the proteasome inhibitor MG132. **(f)** Digoxin dependent splicing inhibition does not depend on the reporter to RBM20 ratio. High concentrations of RBM20 co-transfected with the reporter do not compensate the inhibitory effect. 2-WAY-ANOVA: interaction, column and row factor <0.0001, (N = 4). *P<0.05, ***P<0.001 versus CTRL (Dunnett’s post-test). Data are presented as mean ±SD.

To evaluate the specificity of the splice effect, we generated an independent splice reporter based on formin-like 3 (FMNL3) and its splice factor RBFOX1 (S5 Fig). RBFOX1 and RBM20 have diverse substrate spectra and the respective mutants have different cardiac phenotypes [2,17,18], suggesting that there is no significant interplay between these proteins or its substrates. The FMNL3 splice reporter construct faithfully captures the RBFOX1 splice effect as the RNA and luciferase based analysis produce identical PSI values (S5C vs. S5F Fig). Similar to the titin reporter it responds to increasing concentrations of its splice factor RBFOX1 up to a 100-fold molar excess (S5G Fig). FMNL3 splicing was not affected at digoxin concentrations of up to 20x the half-maximal lethal dose (IC50 viability = 132 nM; IC50 splicing = 2.6 μM), suggesting that there is no primary effect of digoxin on RBFOX1 dependent splicing (Fig 5A). To exclude an unspecific effect of cell death on RBM20 dependent splicing, we used the steroidal homolog digitonin to induce cell death. Digitonin had little effect at the highest doses only but not on the RNA level (Fig 5B). This would suggest that digitonin induced toxicity does not affect alternative splicing, unlike cardenolides. In a complementary approach, we added the general splice inhibitor isoginkgetin and found an effect on RBM20 dependent splicing as well as general mRNA maturation only at the highest toxic dose (30 μM) (Fig 5C). In contrast to the general splicing inhibitor isoginkgetin, which prevents the recruitment of an operative U4/U6.U5 tri-snRNP complex inhibiting basic splicing [19], digoxin only inhibits alternative splicing and does not cause intron retention (S6 Fig).

Towards understanding the molecular basis of cardenolide mediated inhibition of titin splicing, we evaluated its binding to RBM20, the effect on RBM20 protein expression, and on the expression of co-factors, such as components of the spliceosome and RBM20 binding proteins. To evaluate if cardenolides interfere with RNA binding of RBM20, we immobilized human serum albumin (HSA) or the RNA recognition motive (RRM) of RBM20 on Biacore sensor chips, which were incubated with increasing concentrations of digitoxin or digitoxigenin (S7 Fig). Accordingly, we investigated the contribution of cardenolide induced changes in gene expression to the splice effects. At 0.235 μM digoxin RBM20 protein levels were reduced to 10%, an effect that was independent of proteasomal degradation as the proteasome inhibitor MG132 did not rescue RBM20 protein expression (Fig 5D and 5E). Nevertheless, digoxin induced splice repression could not be overcome by elevating RBM20 levels, as increasing amounts of RBM20 expression plasmid did not reconstitute splicing activity at 0.235 μM digoxin (Fig 5F), suggesting that the effect on protein expression alone is not responsible for the splice inhibitory effect.

**Fig 6:**
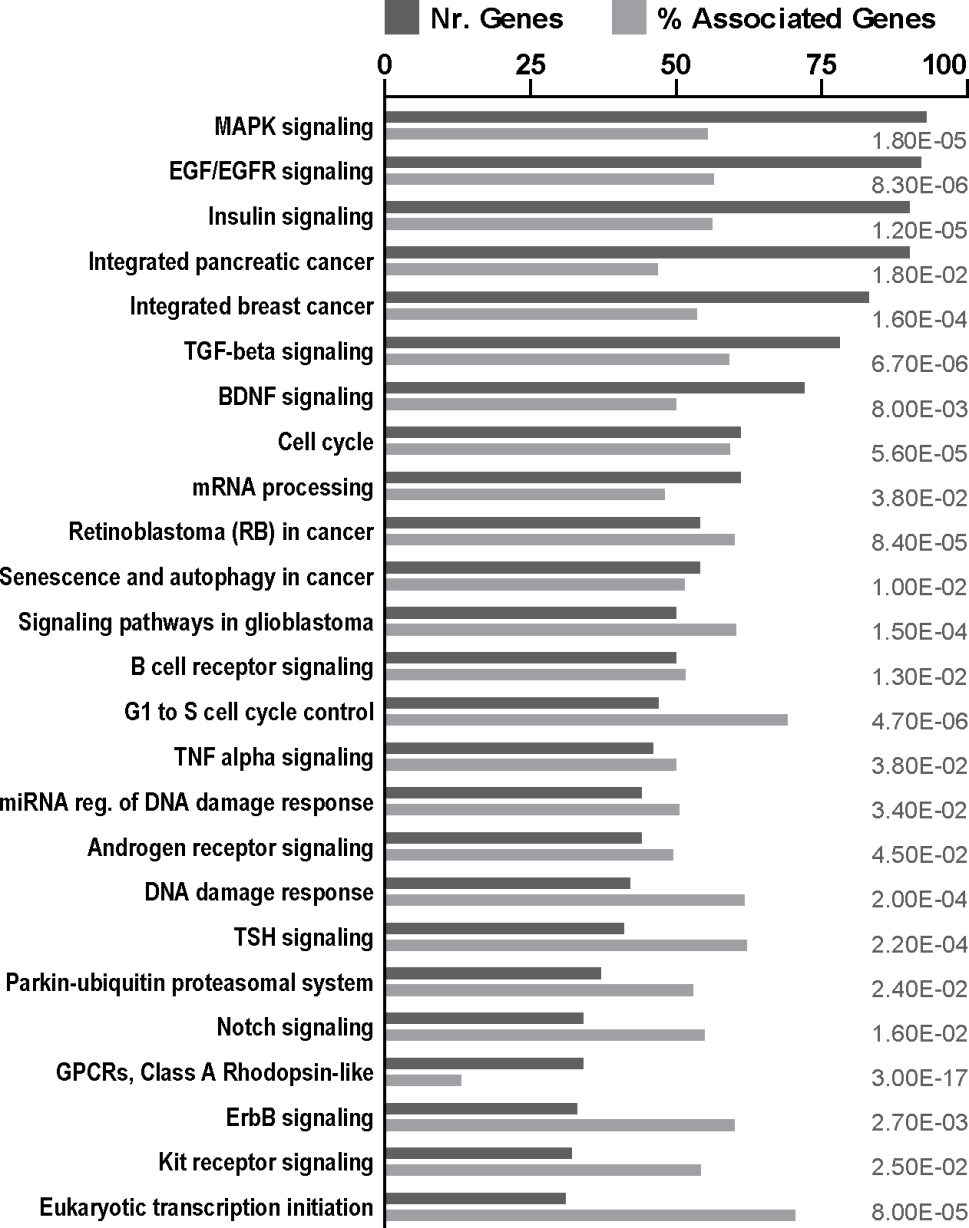
Digitoxin affects mRNA processing and various signaling pathways related to cell division, signal transduction and cancer. Gene ontology according to WikiPathways analysis of transcripts differentially regulated by digitoxin in HEK293 suggest specific effects that include pathways related to growth and cell cycle as well as differential expression of > 60 genes involved in mRNA processing (adjusted *P* ≤ 0.05 [Benjamin-Hochberg], two-sided hypergeometric test).

**Fig 7:**
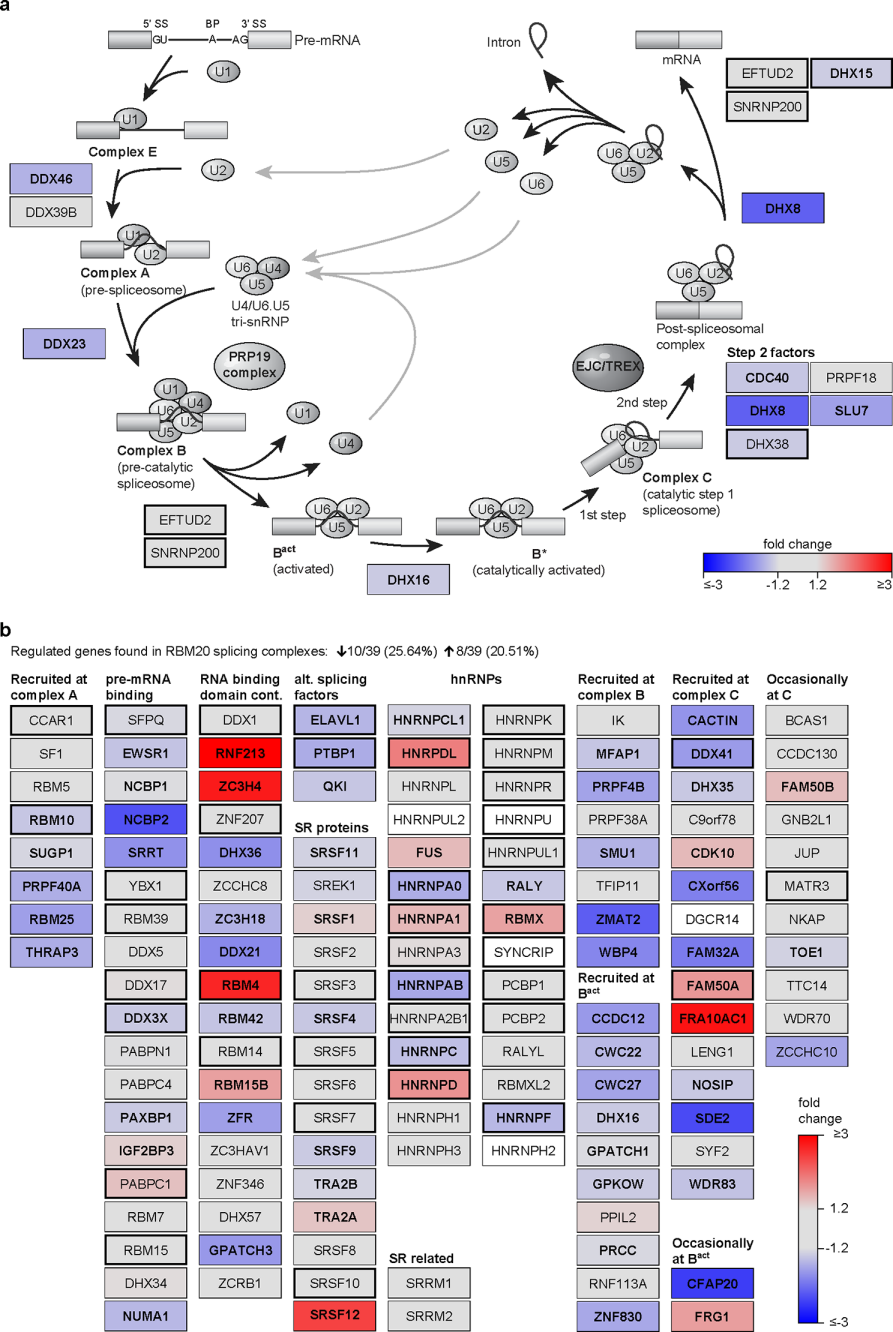
Digitoxin alters transcription of splicing related genes. **(a)** Stepwise assembly of major spliceosomal small nuclear ribonucleo proteins (snRNPs) on pre-mRNA. The removal of non-coding sequence from pre-mature mRNA transcripts requires five RNP complexes U1, U2, U4, U5 and U6. Boxes display the genes related to RNA unwinding and complex remodelling/recycling (DExD/H box proteins) as well as factors necessary for the transesterification. 9 of 12 genes are significantly downregulated by at least 20%. Boxes with thick outlines represent proteins binding RBM20 [22]. **(b)** Target specific factors from human spliceosomal complexes A, B, B^act^ and C that are dynamically integrated into active spliceosomes. Almost half of the genes in RBM20 dependent splicing complexes are significantly regulated by digitoxin.

This prompted us to evaluate the effect of the cardenolides on the RBM20 related splicing network. We analyzed the gene expression profile in HEK293 upon digitoxin treatment (GEO dataset GSE36058 [20]). We found that application of digitoxin affects many cellular processes essential for cell growth and communication, as well as mRNA maturation (Fig 6). The 61 genes associated with mRNA processing encode components of the spliceososomal complexes A, B and C (Fig 7A and S8 Fig) as well as additional factors that relate to alternative splicing [21] (Fig 7B). All significantly regulated components of the core spliceosome, RNA-helicases, and the small nuclear ribonucleoproteins U1-, U2- and the U4/U6.U5 tri-snRNPs are down regulated. Among the 39 proteins previously associated with RBM20 dependent splicing 10 are down and 8 are upregulated suggesting that the alternative expression of RBM20 interacting factors contribute to the effect of cardenolides on alternative splicing.

## Discussion

Diastolic heart failure is notoriously difficult to treat with little or no benefit from classical heart failure medication such as ACE inhibitors, angiotensin receptor blockers, aldosterone antagonists, or beta-blockers [23–25]. Thus, AHA/ACC guidelines focus on risk factors such as hypertension, arrhythmia, increased venous pressure, myocardial ischemia, diabetes and lack of physical fitness, rather than causal treatment [26,27].

The elastic scaffold protein titin is in addition to collagen the main determinant of cardiac filling in diastole [28] and would therefore be a prime therapeutic target for diastolic heart failure. So far there is no titin directed therapeutic approach that would specifically alter titin’s elastic properties–in part because of the complex interplay of its elastic and structural functions that depend on multiple domains and protein/protein interactions [29]. Titin’s elastic properties are adjusted by extensive posttranslational modifications and alternative splicing [30,31]. Posttranslational changes such as phosphorylation are usually short lived and changes in titin phosphorylation can be compensated at the level of isoform expression [32]. Indeed, a rare example of causal treatment has targeted titin through the phosphodiesterase type 5A (PDE5A) inhibitor sildenafil, which increases PKG dependent titin N2B phosphorylation. The resulting decrease in passive tension was documented in human skinned heart muscle strips [33] and restored myocardial stiffness in hypertensive dogs [34]. Nevertheless, it failed to improve cardiac function in patients [35].Accordingly, we chose titin directed alternative splicing as our therapeutic target for diastolic heart failure and developed a cell based assay to identify small molecules that inhibit the recently identified titin splice factor RBM20 [2].

High-throughput library screens using well established *in vitro* assay setups that strive for cost effectiveness and a high degree of positive lead identification are essential tools in the drug discovery process [36]. Here we used a cellular assay based on chemiluminescence rather than fluorescence to avoid problems with compounds that absorb or emit fluorescent light and thus interfere with the readout. As a potential drawback, compounds that act as luciferase substrates can appear as false positives, but they can readily be excluded based on their analogous chemical structure.

We established two independent reporter assays within the titin I-band region and a RBFOX1 dependent FMNL3 reporter that served as a negative control. Validation at the RNA level confirms that they faithfully capture the effect on alternative splicing. Based on its increased sensitivity, we chose the RBM20 dependent PEVK reporter over the I96-98 reporter for optimization to the 384 well format. We improved the signal to noise ratio and optimized transfection efficiencies as well as assay kinetics. The dual luciferase assay not only allowed us to monitor exon inclusion, through the ratio of firefly to renilla luciferase, but also to indirectly evaluate toxicity via the effect on transcription/expression that is reflected in the decreased activity of the renilla luciferase in the constitutive exon [37]. As the co-transfection of RBM20 reduced exon inclusion to intermediate levels, our setup provides the unique opportunity to efficiently identify both inhibitors and enhancers of titin splicing.

In the subsequent small molecule screen we identified cardenolides as potent inhibitors of RBM20 mediated titin splicing. A subgroup—the cardiac glycosides—have been used to treat systolic heart failure based on their effect on cellular Na^+^/K+ and Ca^2+^ ion homeostasis. Cardiac glycosides bind and inhibit the Na^+^/K^+^-ATPase in a dose dependent manner, which translates to increased sarcoplasmic Ca^2+^ and a positive ionotropic effect in the heart [38] and additional effects on cellular signaling. Both the effects on ion homeostasis and signal transduction can occur independent from Na^+^/K^+^-ATPase. The signaling pathways affected by cardiac glycosides are diverse and relate to cell proliferation, differentiation, and apoptosis via ERK1/2 and Src signaling pathways, PI3-kinase and PKB, reactive oxygen species (ROS) and NF-kB [39,40]. This is in line with our finding in digitoxin treated HEK293 cells where MAPK and EGF signaling are the top aspects with most of the genes differentially regulated (Fig 6). Cell cycle and initiation of the mammalian basal transcription machinery are also effected by digitoxin treatment. Among the top ten enriched pathways we also find mRNA processing differentially affected, which includes alternative splicing. In the absence of a direct interaction between cardenolides and RBM20 (S7 Fig), and a global effect on RNA maturation (S6 Fig). Indeed, the cardiac glycoside digitoxin depletes the endogenous splice factors SRSF3 and TRA2B to differentially include exon 10 of MAPT in HEK293 and SHSY-5Y cells, linking cardiac glycoside function to alternative splicing [20,41]. Based on our mechanistic analysis, the effect of cardenolides does likely not relate to a direct interaction with proteins of the splicing machinery, but the expression of a specific set of splicing factors and core spliceosomal components that adapt isoform expression of target genes in a concentration dependent manner [42–44]. Indeed, we found a differential effect on RBM20 vs. RBFOX1 mediated splicing with the respective PEVK vs. FMNL reporter suggesting that cardenolide independent splice factors are involved in the processing of FMNL3 by RBFOX1. This opens a therapeutic window for the use of cardenolides as target-specific splicing modulators e.g. in the treatment of diastolic dysfunction.

Several signaling pathways related to cancer are affected by cardiotonic steroids, which have been used to target various cancer types [39]. Nevertheless, toxicity of such agents–including oleandrin—limit the therapeutic use even for cancer therapy [45]. Cytotoxicity is a main obstacle towards adapting cardenolides for the treatment of diastolic dysfunction, as doses needed to induce the splice effect completely inhibited the Na^+^/K^+^-ATPase in human erythrocytes (Table 1).

**Table 1:** Cardenolide effects on alternative splicing vs. ion homeostasis.

	IC_50_ Splicing HEK293 cells [μM]	IC_50_ Na+/K+ ATPase human erythrocytes [46] [μM]	LD_50_ (Rat) ^a^ [mol/kg]
Digoxin	0.1526	0.00039	4.4721
Digitoxin	0.0560	0.00025	4.4764
Ouabain	1.6673	0.00056	4.7797

^a^ Values derived from The DrugBank database version 5.0

Serum concentrations of 0.5–1.5 ng/ml are sufficient to treat systolic heart failure, whereas concentrations above 2.0 ng/ml (0.026 μM) can cause severe side effects [47]. Towards developing available cardenolides for clinical application, several possibilities exist, including neoglycorandomization [48] or a more targeted approach building on structure function relations derived from our splice active compounds. They share the steroidal core structure with an unsaturated, five-membered butyrolactone ring in C-17 that is required for splice inhibition, since hydrocortisone and digitonin do not interfere with RBM20 dependent splicing. The presence of sugars in C-3 is dispensable for splice inhibition–extending the identification of cardiac glycosides as splice inhibitors [41] to compounds that are not glycosylated. Future cardenolide derivatives might separate toxic from splice inhibitory effects to facilitate their use *in vivo*.

We have established robust assays to identify inhibitors of cardiac splicing in non-cardiac cells. While the use of luciferase facilitates application for high-throughput, each assay has to be validated at the RNA level. Although the assay allows screening for inhibitors and enhancers of alternative splicing, the number of false positives is exceedingly high for splice inhibitors as we found compounds with a structure that resemble luciferase substrates luciferin, which might interfere with luciferase activity rather than splicing. The compounds identified are true positives as validated at the RNA level but as cardenolides have a very limited therapeutic window they will require additional chemical optimization for bioavailability and toxicity, before they can be applied *in vivo*.

We developed the first *in vitro* splice reporter to identify small molecules and leads for the treatment of diastolic heart failure. We demonstrated the benefit of our RBM20 dependent titin based splice reporter in a screen of >34,000 diverse small molecules that identified cardenolides as inhibitors of RBM20 dependent splicing. This heterogeneous group of natural compounds has been in clinical use for the treatment of cardiac arrhythmia and congestive heart failure since > 230 years [49]. Based on their broad effects on ion balance, signaling, and transcription, they have recently been evaluated for the treatment of neurodegeneration [39] and autoimmune disease [50].

Based on our identification and characterization of cardenolides as inhibitors of titin splicing, we suggest them as promising leads to modulate titin’s elastic properties as a targeted approach to treating diastolic heart failure.

## Supporting information

S1 Fig RBM20 targets the PEVK and immunoglobulin region in titin’s I-band *in vivo and in vitro*. **(a)** Endogenous PEVK region of titin containing exons 4-13. Boxes indicate exons. Blue lines represent the isoforms generated with and without RBM20. **(b)** PCR products of alternative transcripts in left ventricles of wildtype (+/+), heterozygote (+/-) and RBM20 deficient (-/-). RBM20 heterozygotes express longer transcripts and homozygotes express only the largest PEVK isoform. The shortest transcript in wildtype hearts contains PEVK exon4, 5 and 13. **(c)** Endogenous immunoglobulin region containing titin I-band exons I96-98. Boxes indicate exons, blue lines mature transcripts generated with and without RBM20. **(d)** PCR products of alternative transcripts produced from the I96-98 minigene by RBM20. Exon I97 in excluded in the presence of RBM20. **(e)** Quantitative PCR to determine the abundance of alternatively spliced exon I97 related to the amount of unspliced product presented as percent spliced in (PSI). RBM20 reduces inclusion of exon I97 by ~70% (N = 3). **(f)** Dual luciferase splicing reporter with firefly luciferase (FLuc) integrated into exon I97 and renilla luciferase (RLuc) downstream of exon I98. The FLuc/RLuc ratio reflects the inclusion of alternative exon I97. **(g)** RBM20 shifts alternative splicing of the dual luciferase reporter (DLR) construct to exclude exon I97. **(h, i)** Quantitative PCR (N = 3) and FLuc/RLuc activity (N = 8) produce dissimilar readouts with decreased sensitivity of the luciferase based assay. ***P<0.001 versus CTRL (Dunnett’s post-test). Error bars are presented as mean ±SD.
(TIF)

S2 Fig Assay transfer to the 384-well format and optimization. **(a)** DNA titration for optimized transfection with a peak of renilla luciferase activity at 45 ng/well (N = 8). **(b)** Cell titration for optimized luciferase readout. Relative light units (RLU) are largely unchanged from 5000 to 9000 HEK293 cells/well (N = 8). **(c)** DMSO as the solvent for the compounds to be screened does not interfere with the DLR-assay readout up to concentrations of 1% (N = 8). **(d)** Hits plate from the pilot screen. About 34,000 compounds were evaluated for their effect on DLR-activity at 10 μM. Values deviating ±3 SD from the mean were used to select compounds for independent validation (N = 1). *P<0.05, ***P<0.001 versus CTRL (Dunnett’s post-test). Error bars are presented as mean ±SD.
(TIF)

S3 Fig Additional cardenolides identified in the HTS. **(a-d)** Cymarol, gitoxigenin, AC1NOXXW and digitoxigenin-3-acetate were identified in the pilot screen and independently validated. After oleandrin, cymarol is the second most potent splice inhibitor identified in our screen. Its effect on titin splicing starts at <100 nM (N = 4).
(TIF)

S4 Fig Testing analogous compounds. Ouabain was selected based on its structural homology to identified inhibitors and suppresses RBM20 mediated titin splicing (N = 4).
(TIF)

S5 Fig A titin independent splicing reporter based on FMNL3 and RBFOX1. **(a)** Minigene of FMNL3 exons 25-26. Boxes indicate exons. Blue lines represent the isoforms generated with and without RBFOX1 (N = 8). **(b)** PCR products of alternative transcripts produced from FMNL3 minigene by RBFOX1. RBFOX1 increases exon 25a inclusion. **(c)** Quantitative PCR of the FMNL3 minigene co-transfected with RBFOX1 presented as percent spliced in. RBFOX1 increases exon 25a inclusion by >3-fold (N = 3). **(d)** FMNL3 F/RLuc splicing reporter. FLuc was integrated in exon 25a and renilla luciferase 3’ of exon 26. **(e, f)** Transcripts of FMNL3 splicing reporter generated by RBFOX1. Co-transfection with RBFOX1 leads to increased exon 25a inclusion as quantified by qPCR and presented as percent spliced in in **f**. **(g)** Responsiveness of FMNL3 dual luciferase splicing reporter to increasing ratios of RBFOX1. Inclusion of FLuc increases constantly with molar ratios of RBFOX1 (N = 8). ***P<0.001 versus CTRL (Dunnett’s post-test). Error bars are presented as mean ±SD.
(TIF)

S6 Fig RNA maturation in cardenolide treated cells. Intron retention in HEK293 cells transfected with RBM20 and the splicing reporter minigene I96-98 and treated with the cardenolide digoxin **(a)** and the general splicing inhibitor isoginkgetin **(b)**. Only isoginkgetin affects mRNA maturation with increasing concentration.
(TIF)

S7 Fig Binding of cardenolides to the RNA recognition motif of RBM20. Digitoxin **(a)** and digitoxigenin **(b)** strongly bind human serum albumin (HSA) with increasing concentrations but not the RNA recognition motif (RRM) of RBM20.
(TIF)

S8 Fig Digitoxin regulates transcription of genes encoding several diverse components of the spliceosome. Bold characters indicate significant changes (P = 0.01). Boxes with thick black outlines indicate proteins that exist in a complex with RBM20 *(12)*. The majority of genes is downregulated by digitoxin (spliceosomal complexes A, B, C and the RNP-complexes). Primarily RNA binding proteins, SR proteins and hnRNPs are differentially regulated in either direction. Gene numbers and percentages are provided above.
(TIF)

S1 Table Primary validation of splice active compounds (cardenolides). (DOCX)

S2 Table Primer sets for RT-qPCR. (DOCX)

S3 Table Primer sets for detection of transcripts from minigene and splicing reporters. (DOCX)
