# Supplementary figures and images for: Drug discovery with an RBM20 dependent titin splice reporter identifies cardenolides as lead structures to improve cardiac filling
Source: PLoS One. 2018 Jun 11;13(6):e0198492. doi: 10.1371/journal.pone.0198492 (PMC5995442; doi:10.1371/journal.pone.0198492)

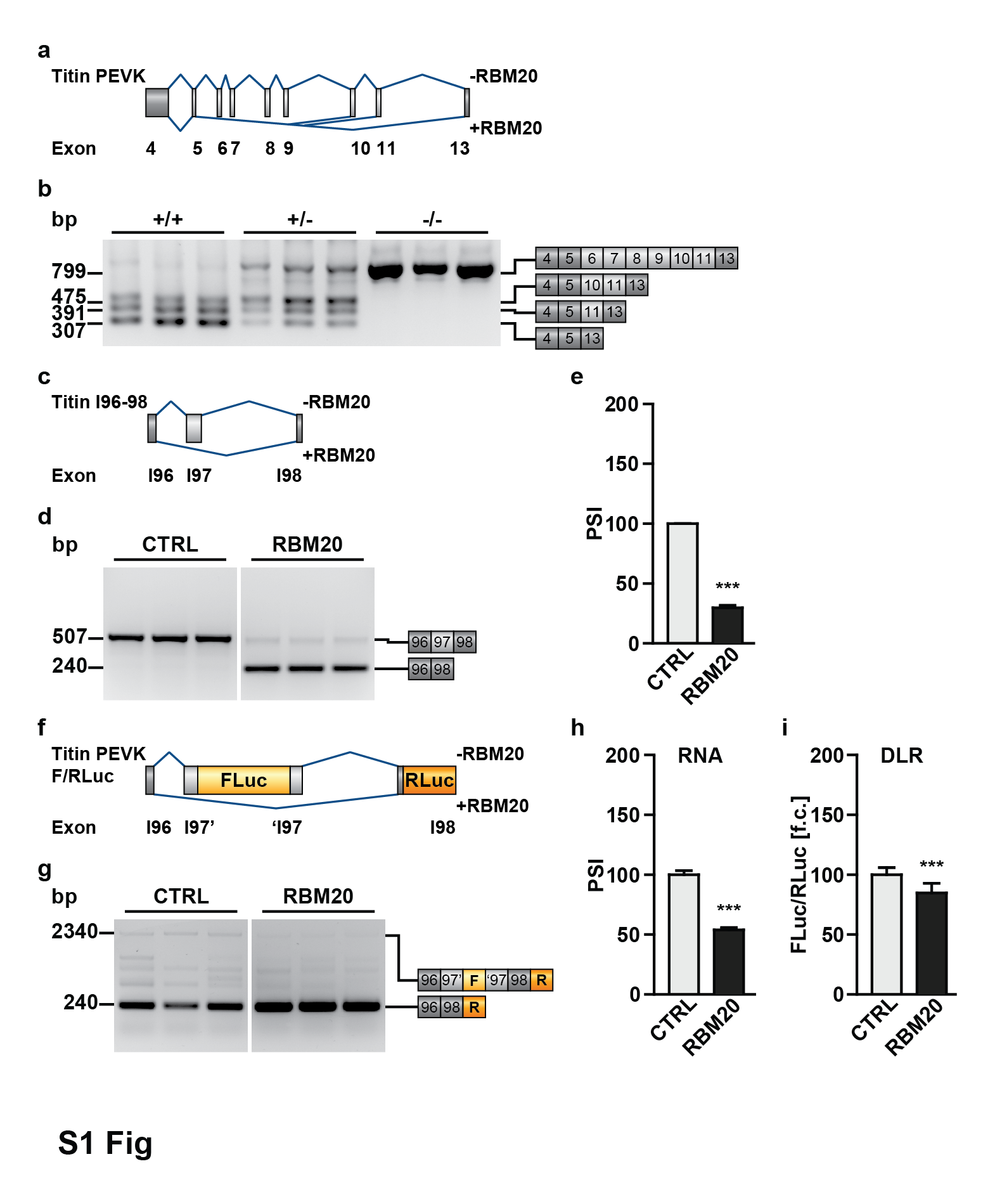

Supplement: S1 Fig — (a) Endogenous PEVK region of titin containing exons 4-13. Boxes indicate exons. Blue lines represent the isoforms generated with and without RBM20. (b) PCR products of alternative transcripts in left ventricles of wildtype (+/+), heterozygote (+/-) and RBM20 deficient (-/-). RBM20 heterozygotes express longer transcripts and homozygotes express only the largest PEVK isoform. The shortest transcript in wildtype hearts contains PEVK exon4, 5 and 13. (c) Endogenous immunoglobulin region containing titin I-band exons I96-98. Boxes indicate exons, blue lines mature transcripts generated with and without RBM20. (d) PCR products of alternative transcripts produced from the I96-98 minigene by RBM20. Exon I97 in excluded in the presence of RBM20. (e) Quantitative PCR to determine the abundance of alternatively spliced exon I97 related to the amount of unspliced product presented as percent spliced in (PSI). RBM20 reduces inclusion of exon I97 by ~70% (N = 3). (f) Dual luciferase splicing reporter with firefly luciferase (FLuc) integrated into exon I97 and renilla luciferase (RLuc) downstream of exon I98. The FLuc/RLuc ratio reflects the inclusion of alternative exon I97. (g) RBM20 shifts alternative splicing of the dual luciferase reporter (DLR) construct to exclude exon I97. (h, i) Quantitative PCR (N = 3) and FLuc/RLuc activity (N = 8) produce dissimilar readouts with decreased sensitivity of the luciferase based assay. ***P<0.001 versus CTRL (Dunnett’s post-test). Error bars are presented as mean ±SD. (TIF) [file pone.0198492.s001.tif]

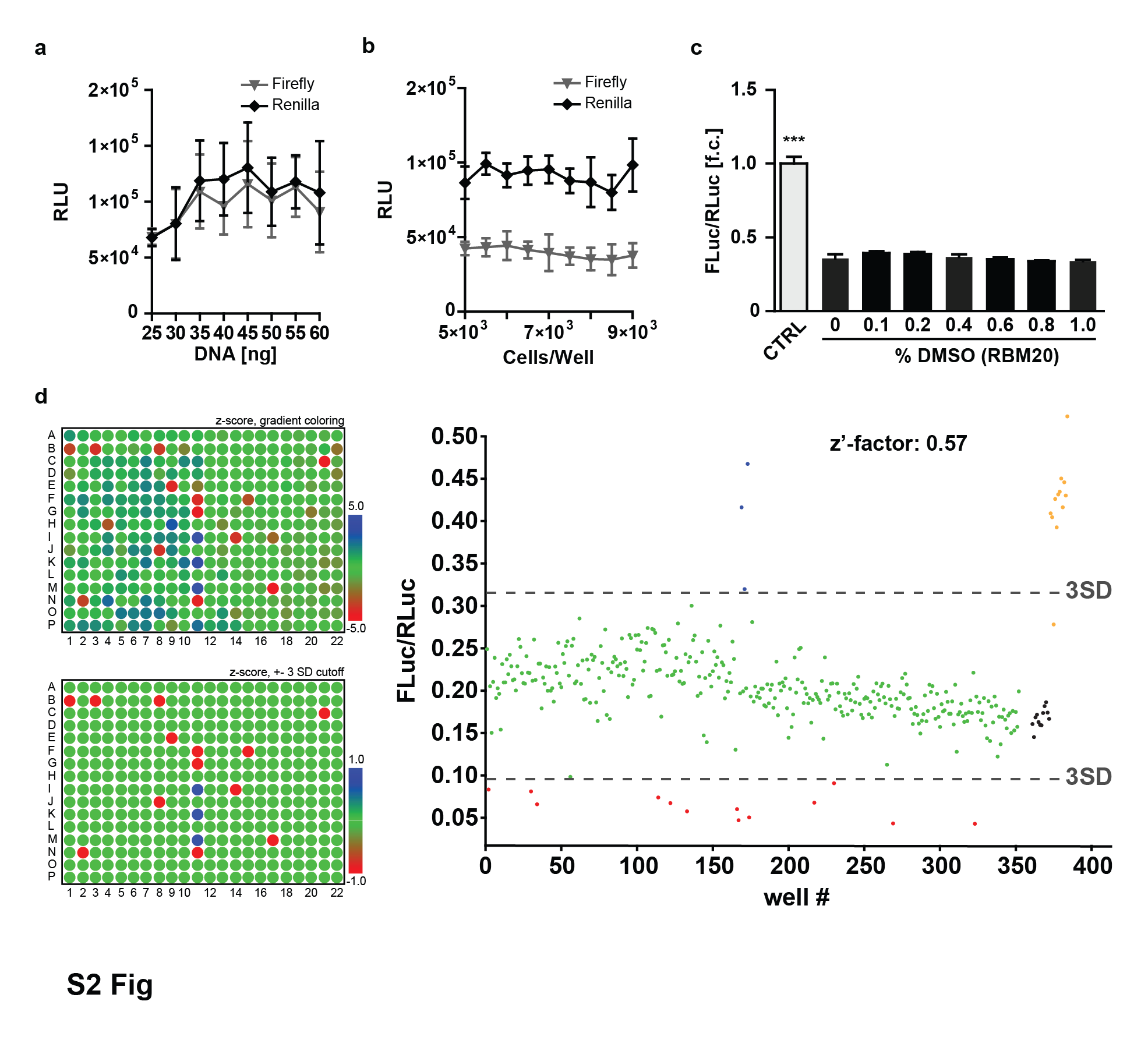

Supplement: S2 Fig — (a) DNA titration for optimized transfection with a peak of renilla luciferase activity at 45 ng/well (N = 8). (b) Cell titration for optimized luciferase readout. Relative light units (RLU) are largely unchanged from 5000 to 9000 HEK293 cells/well (N = 8). (c) DMSO as the solvent for the compounds to be screened does not interfere with the DLR-assay readout up to concentrations of 1% (N = 8). (d) Hits plate from the pilot screen. About 34,000 compounds were evaluated for their effect on DLR-activity at 10 μM. Values deviating ±3 SD from the mean were used to select compounds for independent validation (N = 1). *P<0.05, ***P<0.001 versus CTRL (Dunnett’s post-test). Error bars are presented as mean ±SD. (TIF) [file pone.0198492.s002.tif]

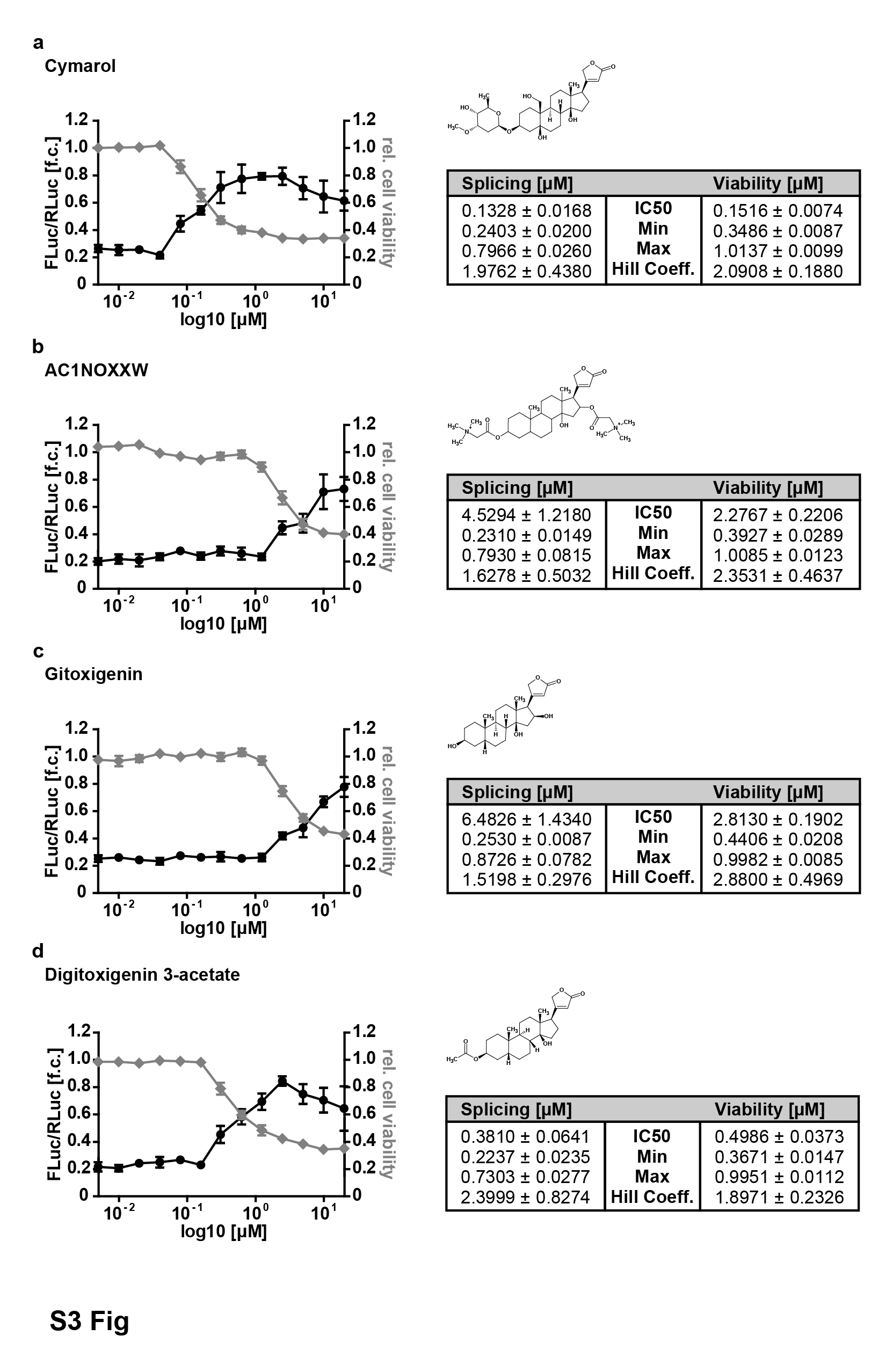

Supplement: S3 Fig — (a-d) Cymarol, gitoxigenin, AC1NOXXW and digitoxigenin-3-acetate were identified in the pilot screen and independently validated. After oleandrin, cymarol is the second most potent splice inhibitor identified in our screen. Its effect on titin splicing starts at <100 nM (N = 4). (TIF) [file pone.0198492.s003.tif]

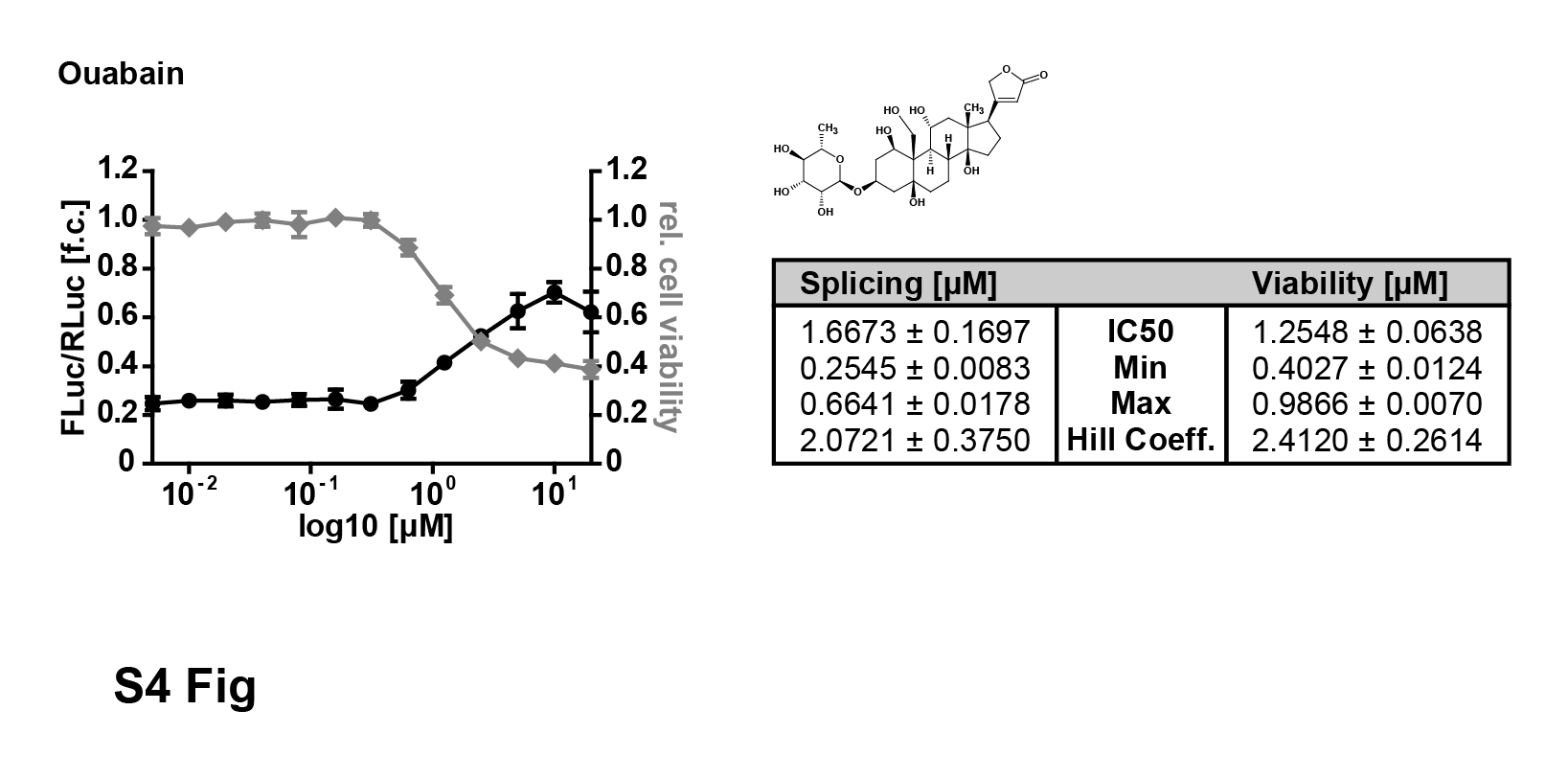

Supplement: S4 Fig — Ouabain was selected based on its structural homology to identified inhibitors and suppresses RBM20 mediated titin splicing (N = 4). (TIF) [file pone.0198492.s004.tif]

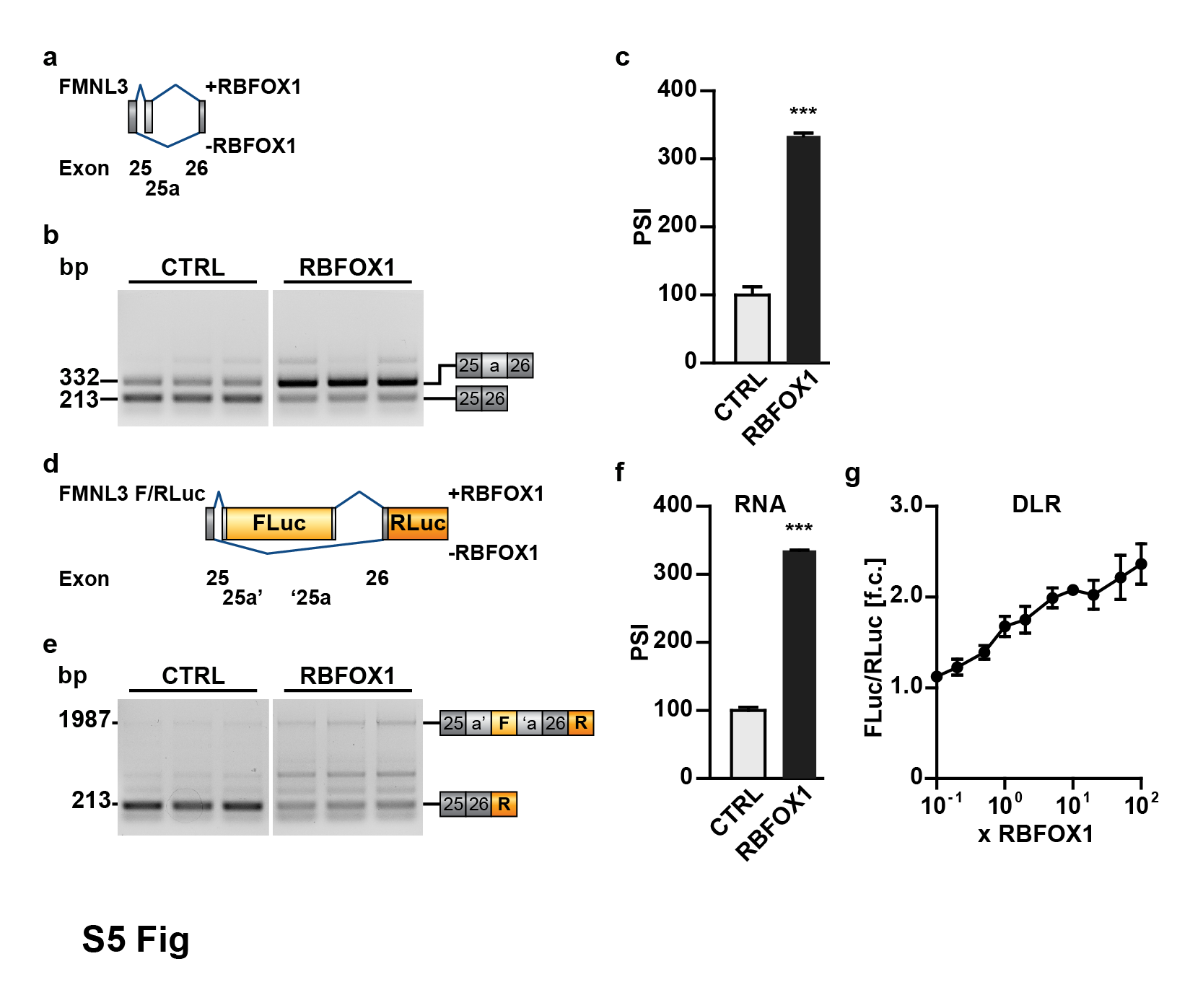

Supplement: S5 Fig — (a) Minigene of FMNL3 exons 25-26. Boxes indicate exons. Blue lines represent the isoforms generated with and without RBFOX1 (N = 8). (b) PCR products of alternative transcripts produced from FMNL3 minigene by RBFOX1. RBFOX1 increases exon 25a inclusion. (c) Quantitative PCR of the FMNL3 minigene co-transfected with RBFOX1 presented as percent spliced in. RBFOX1 increases exon 25a inclusion by >3-fold (N = 3). (d) FMNL3 F/RLuc splicing reporter. FLuc was integrated in exon 25a and renilla luciferase 3’ of exon 26. (e, f) Transcripts of FMNL3 splicing reporter generated by RBFOX1. Co-transfection with RBFOX1 leads to increased exon 25a inclusion as quantified by qPCR and presented as percent spliced in in f. (g) Responsiveness of FMNL3 dual luciferase splicing reporter to increasing ratios of RBFOX1. Inclusion of FLuc increases constantly with molar ratios of RBFOX1 (N = 8). ***P<0.001 versus CTRL (Dunnett’s post-test). Error bars are presented as mean ±SD. (TIF) [file pone.0198492.s005.tif]

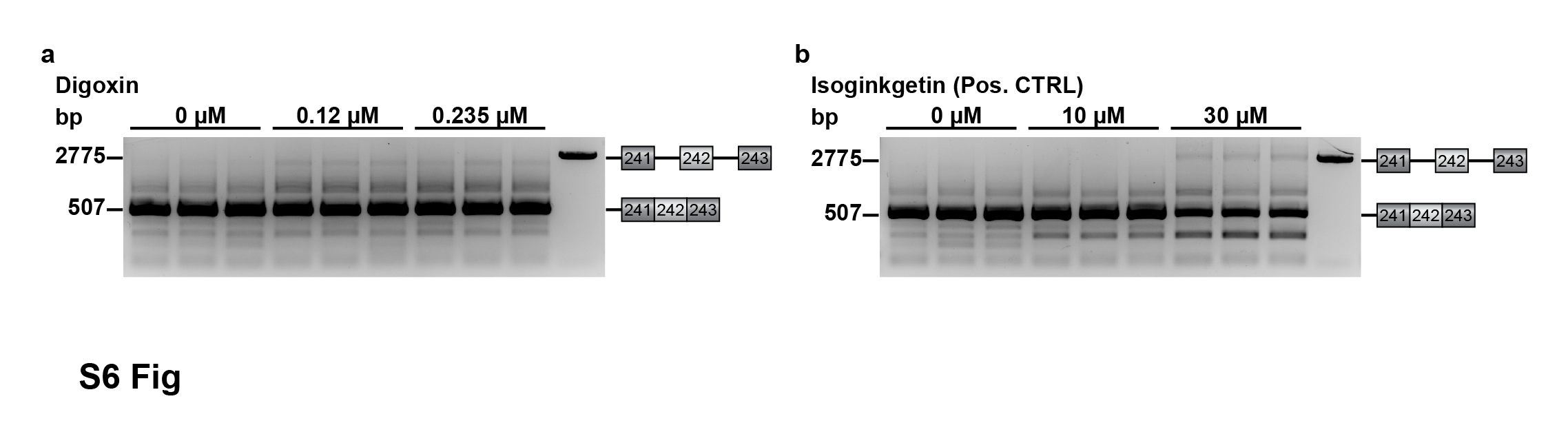

Supplement: S6 Fig — Intron retention in HEK293 cells transfected with RBM20 and the splicing reporter minigene I96-98 and treated with the cardenolide digoxin (a) and the general splicing inhibitor isoginkgetin (b). Only isoginkgetin affects mRNA maturation with increasing concentration. (TIF) [file pone.0198492.s006.tif]

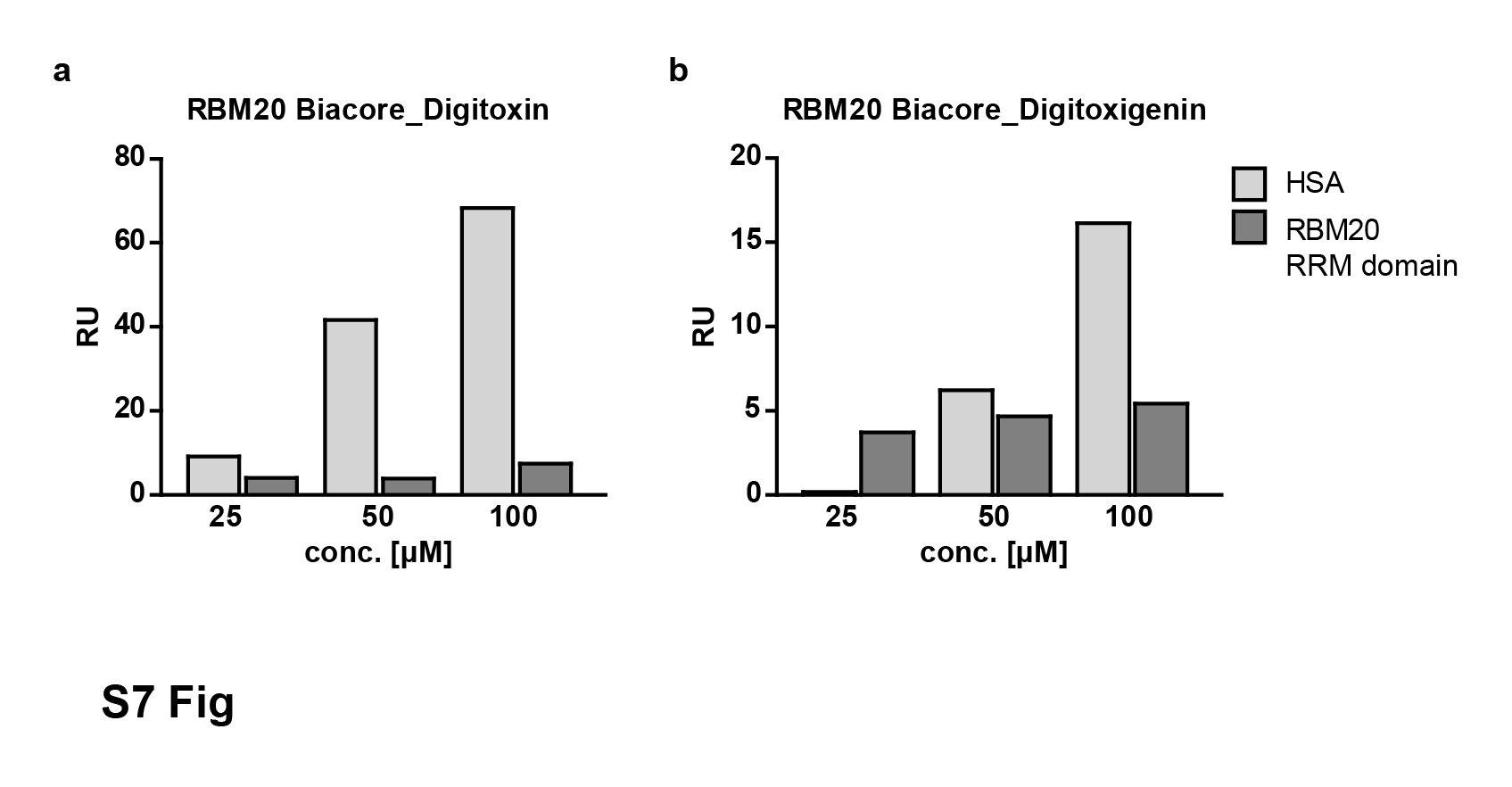

Supplement: S7 Fig — Digitoxin (a) and digitoxigenin (b) strongly bind human serum albumin (HSA) with increasing concentrations but not the RNA recognition motif (RRM) of RBM20. (TIF) [file pone.0198492.s007.tif]

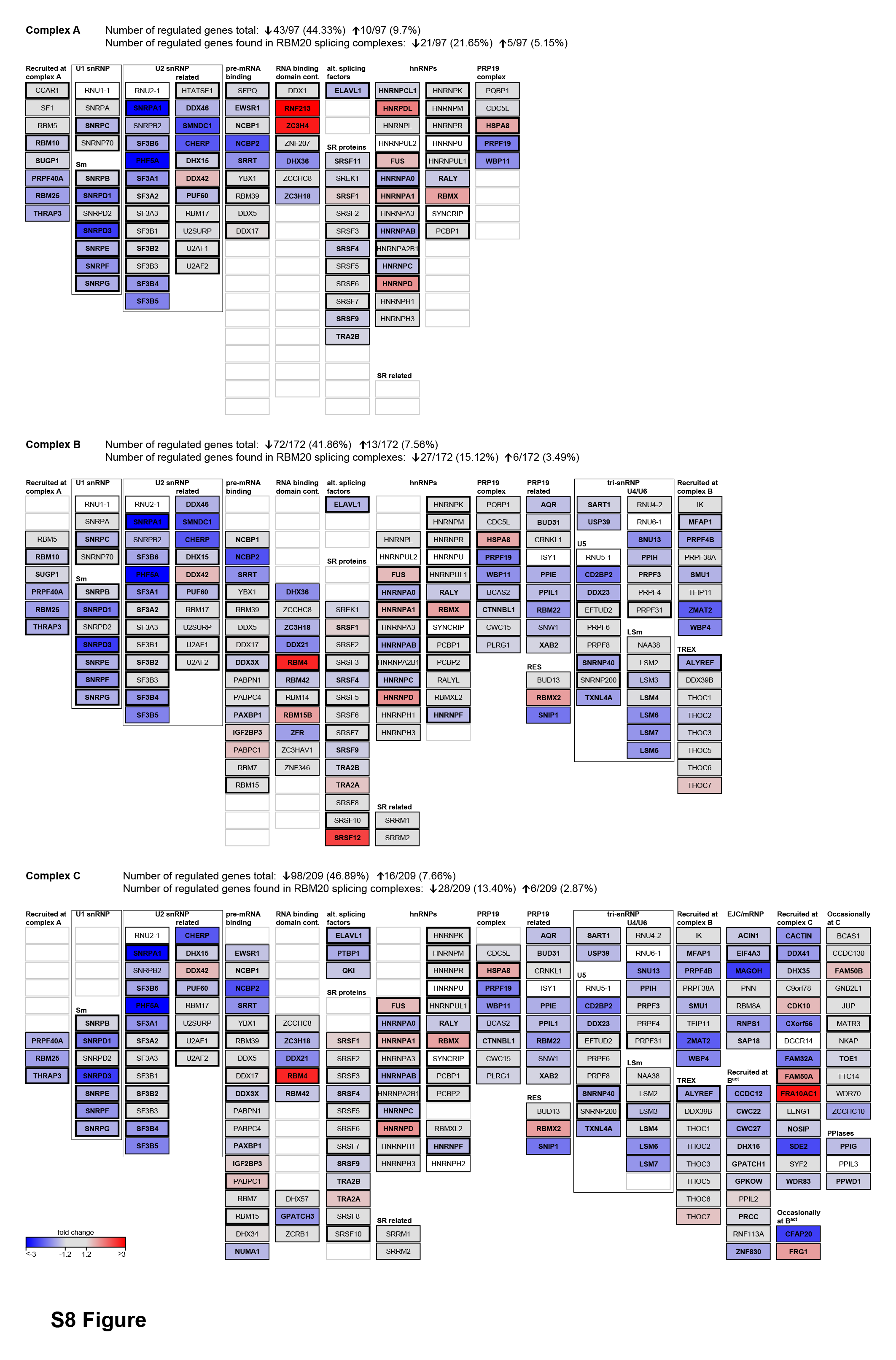

Supplement: S8 Fig — Bold characters indicate significant changes (P = 0.01). Boxes with thick black outlines indicate proteins that exist in a complex with RBM20 (12). The majority of genes is downregulated by digitoxin (spliceosomal complexes A, B, C and the RNP-complexes). Primarily RNA binding proteins, SR proteins and hnRNPs are differentially regulated in either direction. Gene numbers and percentages are provided above. (TIF) [file pone.0198492.s008.tif]
