# Supplementary material for: Drug discovery with an RBM20 dependent titin splice reporter identifies cardenolides as lead structures to improve cardiac filling
Source: PLoS One. 2018 Jun 11;13(6):e0198492. doi: 10.1371/journal.pone.0198492 (PMC5995442; doi:10.1371/journal.pone.0198492)
Supplement: S1 Table — (DOCX) [file pone.0198492.s009.docx]

**S1 Table. Primary validation of splice active compounds (cardenolides).**

| **Compound** | **MW [g/mol]** | **PubChem CID** |
| --- | --- | --- |
| k-Strophanthidol^a^ | 406.51 | 544146 |
| Gitoxigenin | 390.51 | 348482 |
| Digitoxigenin 3-acetate | 416.55 | 10916723 |
| Digoxigenin | 390.51 | 15478 |
| Oleandrin | 576.72 | 11541511 |
| Frugoside^a^ | 536.65 | 120728 |
| AC1NQ5IB^a^ | 448.55 | 5215651 |
| Digitoxigenin | 374.51 | 4369270 |
| Cymarol | 550.68 | 574664 |
| AC1NOXXW | 590.79 | 5096228 |

^a^ not commercially available at the time of investigation
