# Supplementary material for: Drug discovery with an RBM20 dependent titin splice reporter identifies cardenolides as lead structures to improve cardiac filling
Source: PLoS One. 2018 Jun 11;13(6):e0198492. doi: 10.1371/journal.pone.0198492 (PMC5995442; doi:10.1371/journal.pone.0198492)
Supplement: S2 Table — (DOCX) [file pone.0198492.s010.docx]

**S2 Table.** **Primer sets for RT-qPCR.**

| **Name** | **Sequence** | **Target** | **Amplicon [bp]** |
| --- | --- | --- | --- |
| 18S_F | CGCCGCTAGAGGTGAAATTC | 18S | 62 |
| 18S_R | TGGGCAAATGCTTTCGCTC |  |  |
| rRBM20_Ex8f | TGGCTGCTATCATCCAGG | RBM20 | 134 |
| rRBM20_Ex9r | GAGTGATCGGCTCATTGG |  |  |
| hTTN241_SyG2f | CTGAGGGATGTAAAGTTAGAAGATGC | TTN  Ex241-42 (minigene +reporter) | 101 |
| hTTN242_SyGr | AATTCAACTGGGGGTTCTTTCAC |  |  |
| hTTN241_SyG2f | CTGAGGGATGTAAAGTTAGAAGATGC | TTN  Ex241-43  (minigene +reporter) | 102 |
| hTTN243_SyGr | GAATTCCACATGAGGAGCTTTCAC |  |  |
| rPEVK4_SyGf | AGAAGAGGGCTACGATGAAGG | TTN PEVK  Ex4 (minigene +reporter) | 86 |
| rPEVK4_SyGr | CGTGAACCTCCTTCTGAACC |  |  |
| rPEVK8_SyGf | CCAGCTGTGCACACAAAGAAG | TTN PEVK  Ex8 (minigene) | 156 |
| rPEVK8_SyGrn | TGTGACAGACACCTCCTCCTC |  |  |
| FLuc_SyGf | TCAGAGAGATCCTCATAAAGGCC | TTN PEVK  Ex8 (reporter) | 140 |
| rPEVK8_SyGrn | TGTGACAGACACCTCCTCCTC |  |  |
| ivhFMNL3_Ex25f | CAAGCTGGCTAGCATGACC | FMNL3  Ex25-25a (minigene +reporter) | 155 |
| ivhFMNL3_Ex25ar | GACACTCTTCAGCATCTGTGATG |  |  |
| ivhFMNL3_Ex25f | CAAGCTGGCTAGCATGACC | FMNL3  Ex25-26  (minigene +reporter) | 153 |
| ivhFMNL3_Ex26r | GGCAGTGGAGGCCTGTG |  |  |
