# Supplementary material for: Drug discovery with an RBM20 dependent titin splice reporter identifies cardenolides as lead structures to improve cardiac filling
Source: PLoS One. 2018 Jun 11;13(6):e0198492. doi: 10.1371/journal.pone.0198492 (PMC5995442; doi:10.1371/journal.pone.0198492)
Supplement: S3 Table — (DOCX) [file pone.0198492.s011.docx]

**S3 Table.** **Primer sets for detection of transcripts from minigene and splicing reporters.**

| **Name** | **Sequence** | **Target** |
| --- | --- | --- |
| BHKozhTTN241 | CGCGGATCCAAGCTTGCCACCATGGTGGTCCCAC GTTCAGAAG | Human TTN Ex241-243 |
| hTTN 243 XhoI | CCGCTCGAGACTTAGCATTTTCTCGGGAAAGT |  |
| PEVK4_SyGf | AGAAGAGGGCTACGATGAAGG | Rat TTN PEVK Ex4-13 (minigene) |
| PEVKEx13Del30r | CCCTCGAGCAGGAGCAGGTTTCTTTGGAGCC |  |
| PEVKEx4Kfor | CCGGATCCACCATGGAGGAGATCAAGGTGGAAGC | Rat TTN PEVK Ex4-13 (reporter) |
| PEVKEx13rew | CCCTCGAGTCTTCTTTGCCACAGGAACG |  |
| hFMNL3_seqf | CAGGAGTTAATAGCAGAGTTGAGG | Human FMNL3  Ex25-26 (minigene +reporter) |
| hFMNL3_seqr | GTGGCGAACAACCATAGGC |  |
| mouse ttn Ex PEVK4 | GAGCCATATGAAGAACCCTA | Mus TTN PEVK Ex4-13 |
| mPEVK13_r | CTTTAGCTGGAGGGGCTTCC |  |
| rTTN241_f | GTGGTGACACGTTCAGAAGGAAGA | Mus TTN Ex241-243 |
| TTN-243r | CTTTGGAAATTTCGCACTCG |  |
